# Cyclooxygenase-2-Prostaglandin E2 pathway: A key player in tumor-associated immune cells

**DOI:** 10.3389/fonc.2023.1099811

**Published:** 2023-01-27

**Authors:** Kaipeng Jin, Chao Qian, Jinti Lin, Bing Liu

**Affiliations:** ^1^ Department of Orthopedic Surgery, The Second Affiliated Hospital, Zhejiang University School of Medicine, Hangzhou, Zhejiang, China; ^2^ Orthopedics Research Institute of Zhejiang University, Hangzhou, Zhejiang, China; ^3^ Key Laboratory of Motor System Disease Research and Precision Therapy of Zhejiang Province, Hangzhou, Zhejiang, China

**Keywords:** COX-2, PGE2, COX-2 inhibitors, EP, tumor immune escape

## Abstract

Cyclooxygenases-2 (COX-2) and Prostaglandin E2 (PGE2), which are important in chronic inflammatory diseases, can increase tumor incidence and promote tumor growth and metastasis. PGE2 binds to various prostaglandin E receptors to activate specific downstream signaling pathways such as PKA pathway, β-catenin pathway, NF-κB pathway and PI3K/AKT pathway, all of which play important roles in biological and pathological behavior. Nonsteroidal anti-inflammatory drugs (NSAIDs), which play as COX-2 inhibitors, and EP antagonists are important in anti-tumor immune evasion. The COX-2-PGE2 pathway promotes tumor immune evasion by regulating myeloid-derived suppressor cells, lymphocytes (CD8^+^ T cells, CD4^+^ T cells and natural killer cells), and antigen presenting cells (macrophages and dendritic cells). Based on conventional treatment, the addition of COX-2 inhibitors or EP antagonists may enhance immunotherapy response in anti-tumor immune escape. However, there are still a lot of challenges in cancer immunotherapy. In this review, we focus on how the COX-2-PGE2 pathway affects tumor-associated immune cells.

## Introduction

Cyclooxygenases-2 (COX-2) and Prostaglandin E2 (PGE2) are important inflammatory factors, associated with survival, invasion, growth and immune escape of cancer cells. One of the “hallmarks” of cancer is chronic inflammatory disease, which often promotes tumorigenesis and tumor progression (1). For example, inflammatory bowel disease patients had a higher lifetime risk of colon cancer linked to colitis at a younger age than the general population, demonstrating that cancer is easily caused by chronic inflammation (2). Tumor-associated inflammation involves complex interactions between epithelial and mesenchymal cells and in some cases can lead to epigenetic alterations. More broadly, however, chronic inflammation can lead to the production of growth factors that support the development of emerging tumors and cause them to behave as “unhealable wounds” (3). Chronic inflammation can also promote tumor development by facilitating tumor immune escape and establishing an immunosuppressive microenvironment, both of which are cancer-related characteristics. Tumor immune escape occurs through a variety of immunosuppressive mechanisms, such as dysfunctional antigen-presenting cells (APCs), tumor cell resistance to immune attack, decreased cytotoxicity of CD8^+^ T cells and natural killer (NK) cells, induction of immunosuppressive cells such as myeloid-derived suppressor cells (MDSCs), transition of T helper (Th) cells from Th1 to Th2 and Th17, transition of macrophages from M1 to M2 (4).

Numerous studies have shown the importance of nonsteroidal anti-inflammatory drugs (NSAIDs) and EP receptor antagonists in reducing tumor incidence, metastasis and mortality. NSAIDs cause tumor regression and suppress tumor growth by inhibiting the COX-2-PGE2 signaling pathway in several ways: 1. activation of tumor epithelial cells; 2. inhibition of tumor epithelial cell survival and tumor immune surveillance; 3. establishment of tumor-supporting microenvironment; 4. alteration of DNA methylation mechanisms (2). However, the molecular mechanisms underlying the anti-tumor effects of NSAIDs and EP receptor antagonists have not yet been fully understood. More clinical trials and studies are required to explore this further.

In this review, we emphasize our current understanding of COX-2 and PGE2 regulation of tumor immunity. In addition, we are looking into how the COX-2-PGE2 pathway affects tumor-associated immune cells. Given the significance of this pathway in tumor immune escape, we will discuss how to target each component of this pathway as potential strategy for overcoming tumor immune escape while avoiding some serious adverse effects associated with the use of NSAIDs or COX-2 inhibitors.

## Cyclooxygenase

Cyclooxygenases (COX) include COX-1 and COX-2, also known as prostaglandin G/H synthase-1 and -2, are membrane-bound enzymes that are mainly found on the nuclear membrane and luminal side of the endoplasmic reticulum (5). COX-1 is structurally expressed in many healthy tissues and provides steady-state levels of prostaglandins to perform “housekeeping functions”. In contrast, COX-2 is an inducible isoform produced by prostaglandin-like substances that is typically absent or expressed at low levels in normal organs and tissues, but is overexpressed in many tumor and inflammatory tissues (6). In addition to inducing inflammation, COX-2 can also promote cell survival and proliferation. Therefore, COX-2 may be associated with tumorigenesis and development. Indeed, COX-2 has been found to be highly expressed in different tumors, including breast cancer (7), the melanoma (8), and colorectal cancer (2). Its levels are associated with the development, aggressiveness and prognosis of many tumor entities. Regular use of NSAIDs dramatically decreased the incidence of sporadic colorectal cancer as well as breast, lung, and prostate cancers, according to a new analysis that searched all epidemiological studies (case-control and cohort studies) conducted since 1980 (9). According to one study, genetically removing COX enzymes from mouse melanoma (BRAFV600E or NrasG12D), colorectal cancer (CT26) or breast cancer (4T1) cell lines resulted in dramatic tumor eradication (10). When metastases were assessed 2 weeks after surgical resection of the primary breast cancer tumor, fibroblast-targeted Ptgs2-deficient mice (Ptgs2DFb) were found to develop fewer metastases than WT mice (11). Patients with gastric cancer who have low staging and higher levels of COX-2 expression are at greater risk of dying from the disease. In addition, COX-2 has been identified as an independent prognostic factor for gastric cancer (12). According to the findings mentioned above, COX-2 is thought to be crucial for tumor growth.

## COX-2-PGE2 signaling in tumor cells

**Figure 1:**
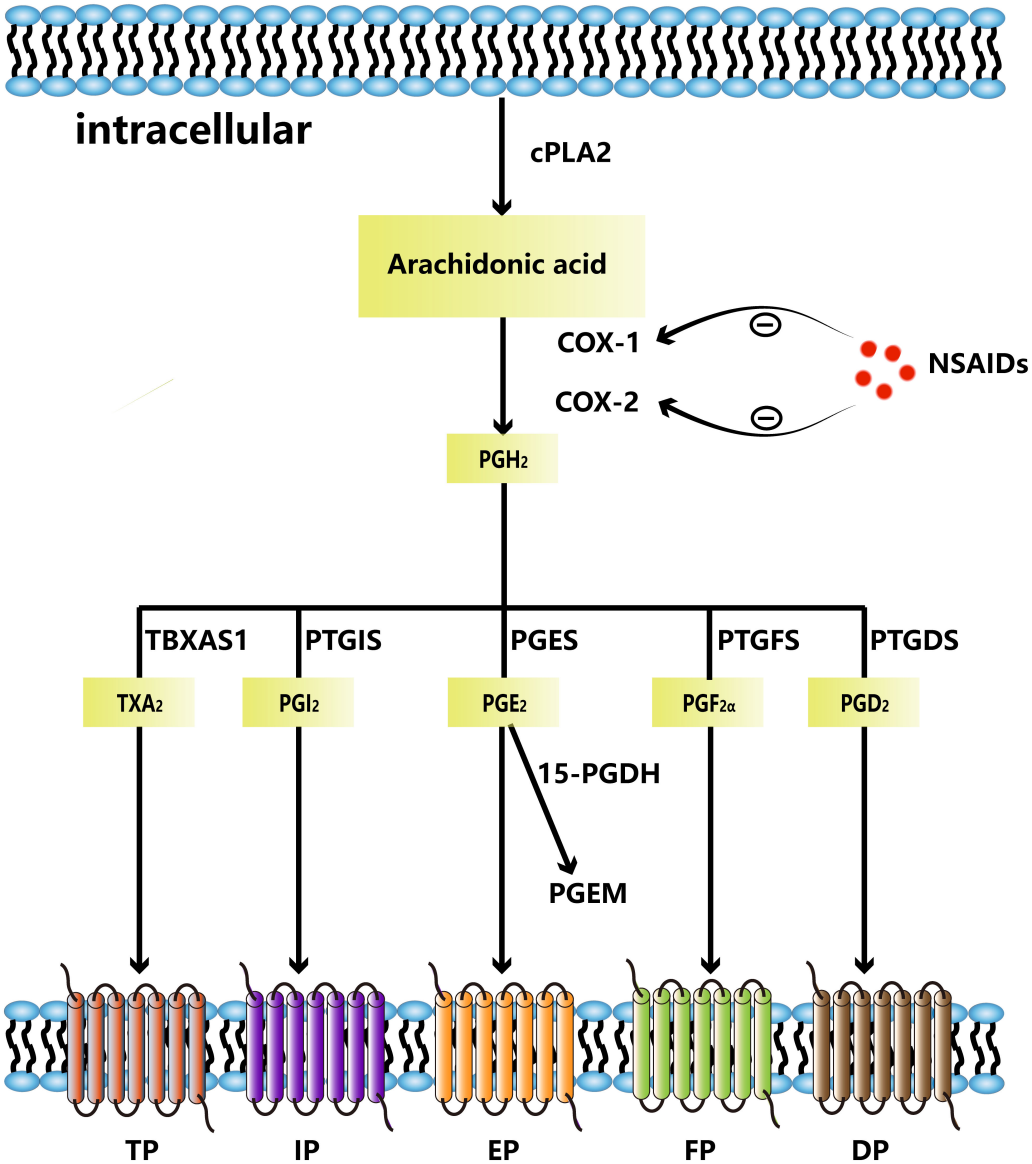
Arachidonic acid is metabolized to PGH_2_ by COX-1 and COX-2. NSAIDs can inhibit COX-1 and COX-2. PGH_2_ is metabolized to thromboxane A2(TXA_2_)by TBXAS1, PGI_2_ by PTGIS, PGE_2_ by PGES, PGF_2α_ by PGES, PGD_2_ by PTGDS, respectively. PGE_2_ can be metabolized to PGEM by 15-PDGH. TXA_2_ binds to TP, PGI_2_ binds to IP, PGE_2_ binds to EP, PGF_2α_ binds to FP and PGD_2_ binds to DP.

COX enzymes convert arachidonic acid to the endogenous peroxidation intermediate PGH2, which is modified by prostaglandin synthase to produce five structurally related prostaglandins, including prostaglandin D2, prostaglandin E2, prostaglandin I2, prostaglandin F2α, and thromboxane A2. The two essential enzymes that catalyze the initial and concluding phases of this synthetic pathway are COX-2 and prostaglandin E2 synthase (PGES), including microsomal PGES (mPGES) and cytosolic PGES. In addition, PGE2 is converted to its inactive form, 15-keto-PGE2 (PGEM), which is then further metabolized to a stable terminal metabolite by the enzyme 15-hydroxyprostaglandin dehydrogenase (15-PGDH). High levels of PGEM increase the risk of gastric and colorectal cancers (13, 14), implying that PGEM may be a biomolecular marker for cancer risk prediction. Prostaglandins control cellular processes by attaching to G protein-coupled receptors on the cell surface. These cell surface receptors are named EP (EP1, EP2, EP3, EP4), DP (DP1, DP2), FP, IP, and TP. PGE2 transmits signals by binding to four receptors, EP1 through EP4 (15) (**Figure 1**). The expression of each EP receptor and the strength of each EP signal determine the PGE2 signal’s final output. Each EP interacts with its unique G protein to activate particular downstream signaling pathways such as PKA pathway, β-catenin pathway, NF-κB pathway and PI3K/AKT pathway, which have various functions in biological and pathological behavior. Activated EP1 can upregulate the level of intracellular calcium ion concentrations; EP2 and EP4 receptors are associated with cAMP stimulation and PKA signaling through sequential activation of Gas and adenylyl cyclase; EP3 is responsible for downregulating cAMP levels and lead to different cellular responses through different G proteins (16, 17).

**Figure 2:**
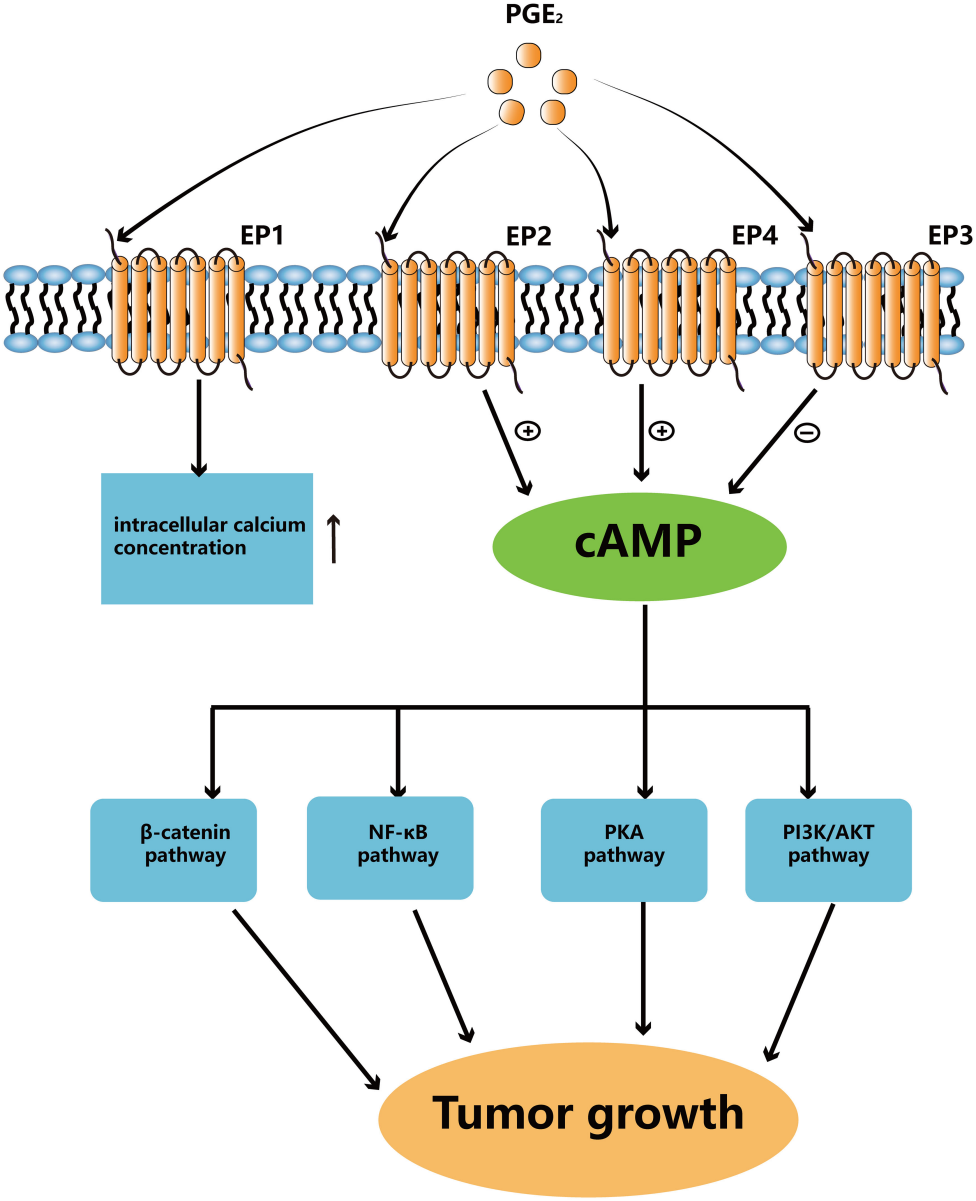
PGE_2_ binds to different EPs to activate downstream signaling pathways. Activated EP1 can upregulate the level of intracellular calcium ion concentrations. EP2 and EP4 can upregulate cAMP levels to activate different pathways which can promote tumor growth. EP3 is responsible for downregulating cAMP levels.

The most abundant prostaglandin, PGE2, is frequently linked to a poor prognosis in a number of human cancers, including colon, head and neck, lung, and breast cancers (6, 17, 18). In immunologically active hosts, the formation of tumors by mutant BRAFV600E mouse melanoma cells requires the synthesis of prostaglandin E2, which inhibits immunity and fosters tumor inflammation (10). It has been shown that mPGES1 expression increased in human melanoma, and that elevated expression of this protein are linked to reduced patient survival (19). The mPGES-1 gene deletion significantly reduced the risk of developing colon cancer and resulted in reduced multiplicity of distal colon tumors as well as tumor load in mice treated with azoxymethane (AOM) (20). In addition, in ApcMin/+ and AOM mice models, elevated endogenous PGE2, caused by 15-PGDH gene deletion, encouraged the development of colon cancer (21). Taken together, PGE2 is essential in the growth of cancers (**Figure 2**).

In addition, numerous evidence has shown that the COX-2-PGE2 pathway promotes tumor development. It has been shown that PGE2 promotes sporadic or colitis-associated colon carcinogenesis *via* EP1 and EP2 receptor in colon cancer cell lines (22). According to research, numerous tumors are linked to EP1 receptor. For example, selective EP1 antagonist (ONO-8713) can significantly reduce the number of tumor cells in mouse with UVB-induced acute skin inflammation (23). PGE2 upregulates anti-apoptosis protein expression in hepatocellular carcinoma cells *via* EP1 receptors (24). While EP3 receptors were downregulated, EP1, EP2 and EP4 receptors were all highly increased in COX-2-driven mammary cancers. Downregulation of EP3 receptors suggests a possible protective role against mammary tumors. EP2 and EP4 receptor levels were reduced in the mammary glands of anti-inflammatory pain-treated mice, while EP1 and EP3 levels were not altered (25). According to Fujino et al, EP3 receptors could boost vascular endothelial growth factor receptor-1 signaling and encourage tumor cell metastasis (26). Therefore, it may be debatable how EP3 contributes to carcinogenesis, and more research is required. The role of the EP4 receptor in tumors appears to be more clearly defined than that of other EP receptors. EP4 is a high-affinity EP receptor, and it is considered to be a pro-cancer mediator in many different types of malignancies due to its high expression. In colorectal cancer, deletion of EP4 can attenuates the abnormal AOM-induced crypt (27, 28), and therefore EP4 receptor can be used for prostate cancer immunotherapy. For example, YY001, an antagonist of the EP4 receptor, inhibits prostate cancer growth by modulating the tumor microenvironment (TME), leading to significant tumor regression, long-term survival and long-lasting immune memory (29). Besides, in mouse model, EP4 knockdown suppressed metastasis of oral cancer cells in the lung (30). Collectively, these findings imply a critical function for the PGE2/EP4 signaling pathway in tumor development.

## COX-2-PGE2 Pathway in Tumor Immunology

**Figure 3:**
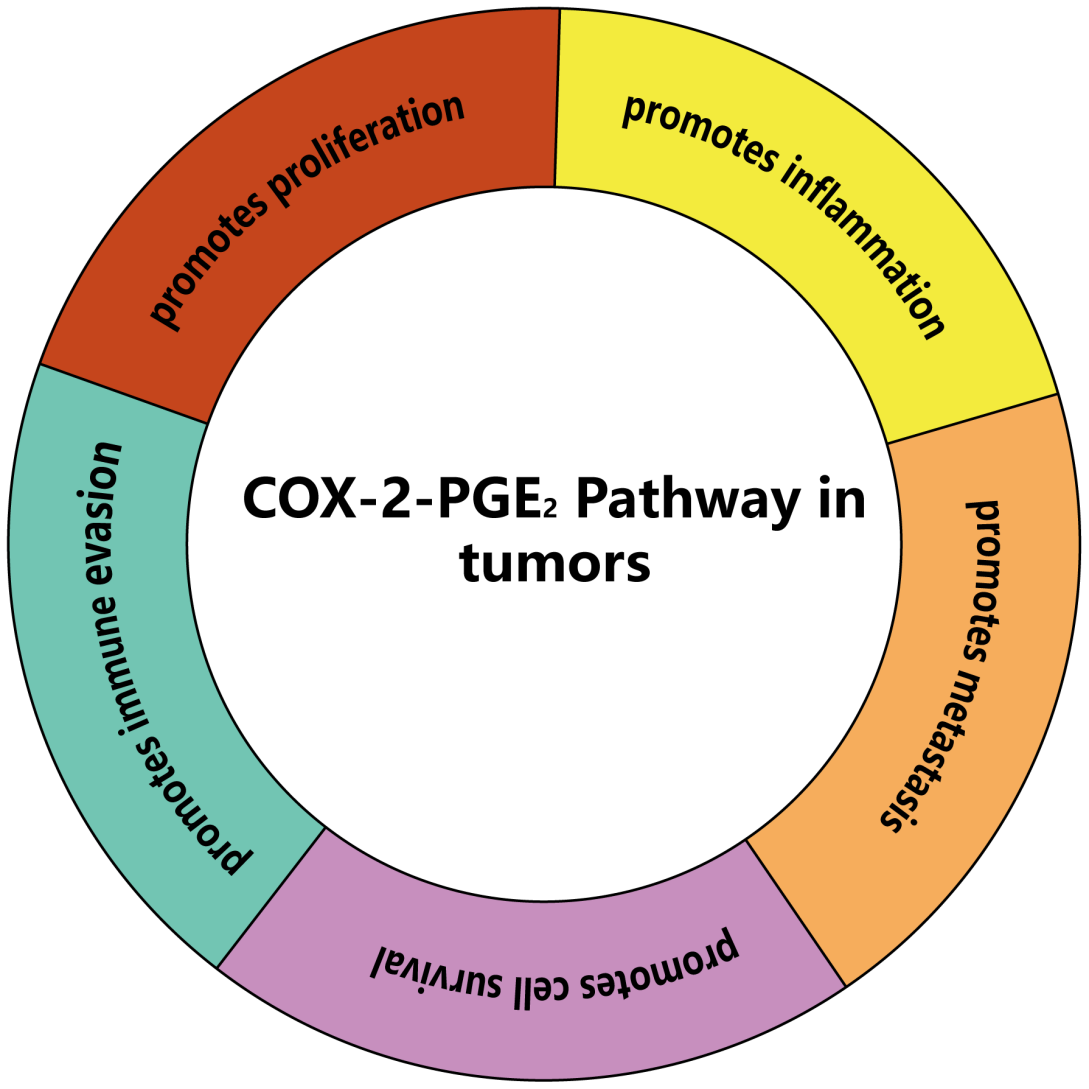
The main effects of COX-2-PGE2 pathway on tumors. COX-2-PGE2 pathway can promote proliferation, inflammation, metastasis, cell survival and immune evasion of tumors.

The COX-2-PGE2 pathway induces tumor immune evasion by regulating myeloid-derived suppressor cells (MDSCs), lymphocytes (CD8^+^ T cells, CD4^+^ T cells and natural killer cells), and antigen presenting cells (APCs). Understanding the mechanisms of the COX-2-PGE2 pathway may provide a solid foundation for developing new methods to overcome tumor immune escape (**Figure 3**).

### Myeloid-derived suppressor cells

**Figure 4:**
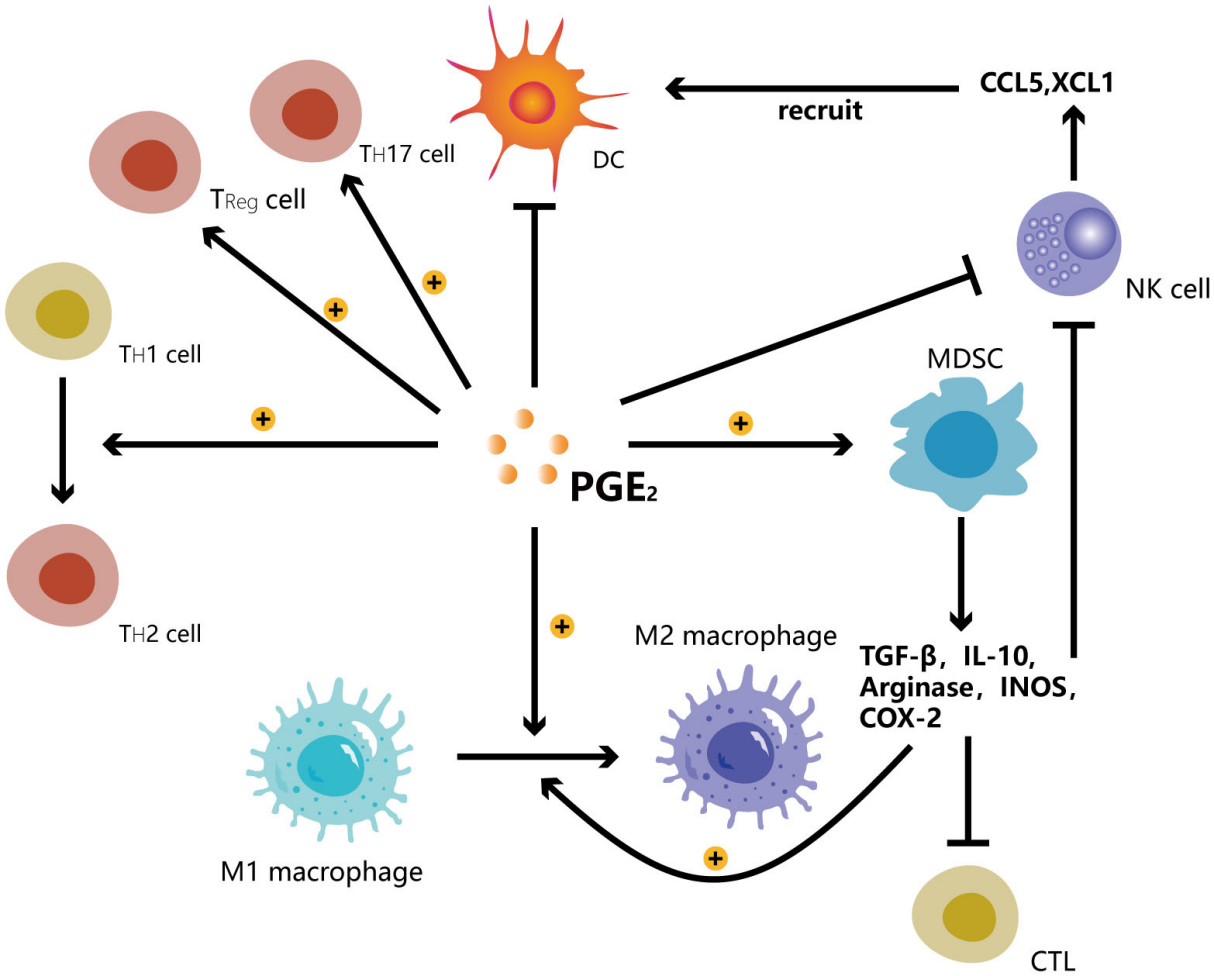
PGE_2_ promotes the transition of T helper cells from TH1 to TH2 and the shift of macrophages from M1 to M2. PGE_2_ induces the development of Treg cells, TH17 cells and MDSCs while inhibiting Dendritic cells, NK cells. MDSCs secrete TGF-β, IL-10, arginase, INOS, COX-2 to promote the shift of macrophages from M1 to M2 while inhibiting the functions of cytotoxic lymphocytes (CTL) and NK cells. NK cells can secrete CCL5 and XCL1 chemokines to recruit DCs.

Granulocyte/polymorphonuclear stem cells and monocyte MDSCs, which come from the granulocyte or monocyte myeloid lineage, respectively, are the two main kinds of MDSCs found in both humans and animals. However, unlike mice, a tiny group of myeloid precursor cells known as “early MDSCs” have been found in humans. These cells have a potent immunosuppressive function and consist mainly of myeloid progenitor cells and precursor cells, accounting for less than 5% of the total number of MDSCs (31). MDSCs mediate immunosuppression by producing arginase and inducible nitric oxide synthase, and TGF-β, IL-10 and COX-2. These substances directly or indirectly enhance Treg activity (32), inhibit cytotoxic activity of NK cells (33), and promote the polarization of macrophages toward M2-like phenotype. They synergistically impair the tumoricidal properties of effector CD8^+^ T cells, leading to tumor cell evasion of host antitumor immunity (34). MDSCs are significantly linked to a poor clinical course of cancer (35). In mice and patients, the clinical cancer stage and the amount of metastatic tumors are favorably linked with the blood levels of MDSCs (36) (**Figure 4**).

The EP4 antagonist YY001 promotes the proliferation and anti-cancer activities of T lymphocytes while inhibiting myeloid-derived suppressor cells’ development, maturation, and immune-suppressive actions. Additionally, it alters the chemokine profile of tumor cells *in vitro* and *in vivo*, which decreases the amounts of MDSCs and T cells in the TME (29). *In vitro* experiments, PGE2 participated in MDSC-mediated immunosuppression by inducing arginase I expression through EP4 and inhibiting effector T cell activity (37). One study found that treatment of colorectal cancer mice with aspirin or EP antagonists significantly protected mice from tumor formation and reduced aggregation of MDSCs and expression of COX-2/Arg-1 (38). Thus, PGE2 inhibits MDSCs by blocking EP4 receptors, thereby promoting tumor immune evasion. The number of Gr1(+) CD11b(+) immature myeloid suppressor cells was considerably decreased by the celecoxib during the chemoprevention of 1,2-dimethylhydrazine diHCL- (1,2-DMH-) induced colon cancers in Swiss mice (39). The COX-2 pathway promotes glioma formation by directly supporting the phylogeny of MDSCs and their accumulation in TME, where MDSCs limit CTL infiltration (40). Taken together, the COX-2-PGE2 signaling pathway promotes tumor immune evasion by inducing the accumulation of MDSCs cells, which in turn promotes tumor growth and development. However, the regulatory role of COX-2-PGE2 pathway on MDSCs *in vivo* has not been fully investigated.

### Macrophages

Microglia, Kupffer cells, and Langerhans cells in the brain, liver, and epidermis are examples of tissue-specific macrophages. Macrophages possess the ability to remove apoptotic cells, cellular debris and pathogens (41). Similar to the Th1 and Th2 dichotomy, the distinction between M1 and M2 phenotypes supports the idea of macrophage phenotypic heterogeneity. When exposed to lipopolysaccharide and interferon-c, macrophages develop the M1 phenotype and exhibit anticancer properties. Macrophages become polarized to the M2 phenotype in response to Th2 cytokines which promote cell proliferation and tumor formation (42, 43). TAMs have an M2-like phenotype in the majority of cancers (44), which facilitates tumor-associated angiogenesis, promotes tumor cell invasion and migration, and inhibits immune surveillance to promote tumor metastasis (45). Clinical studies have shown that macrophages promote tumorigenesis. In a meta-analysis, more than 80% of studies indicated a link between high macrophage density and poor patient prognosis (46). In primary tumors, TAMs have been shown to inhibit CD8^+^ T cell recruitment and anti-tumor immunity (47).

Previous studies have pointed out that the COX-2-PGE2 signaling pathway contributes significantly to the polarization of M1 to M2 and tumor immune escape. The majority of the COX-2 seen in both human and mouse intestinal cancers is known to come from TAMs. Celecoxib, a selective COX-2 inhibitor, shifted the TAM phenotype from M2 to M1 in a colorectal cancer animal model in accordance to the decrease in the number of polyps in ApcMin/1 mice (48). While inhibiting the growth of immune-stimulated M1 macrophages, PGE2-binding EP4 promoted development of immune-suppressed M2 macrophages and MDSCs (49). Eruslanov et al. found that in the mouse colon cancer cell line CT26, overexpression of 15-PGDH shifted TAMs from M2-directed TAM to M1-directed macrophages (50). In the GC transgenic model, overexpression of COX-2 and mPGES-1 led to TAM recruitment in gastric tumors (51). These findings collectively imply that COX-2 and PGE2 stimulate tumor development *via* M2 TAMs. EP1 and EP3 receptors also play an important role in tumor growth. Targeting EP1 receptor can decrease F4/80 (+) macrophage infiltration and inhibit colon cancer growth (52). EP3 signaling in DCs induces a switch of macrophages from pro-inflammatory to pro-reparative phenotype (53).

### NK Cells

As a part of innate immunity, NK cells play an important role in tumor immune surveillance and viral infection resistances. In addition to immediately identifying and eliminating tumor cells, NK cells secrete cytokines that promote CTL activation and growth. However, the functional response of NK cells is compromised in tumors (54). In all cancer types, patient survival was substantially correlated with high expression of NK marker genes (55). In cancerous environment, NK cells dysfunction is integral and inevitable, leading to not only the proliferation of tumor cells but also the formation of distant metastases (56). The abundance of peritumoral NK cells is also associated with high pathological complete response rates in neoadjuvant chemotherapy for large and locally advanced breast cancers (57). In the course of infection with chronic lymphocytic choriomeningitis virus, intrinsic NK cell signaling can inhibit the expansion of CD8^+^ T cells, thereby promoting tumor immune evasion (58).

The COX-2-PGE2 pathway can enhance tumor immune evasion. PGE2 has been reported to inhibit cytotoxic effects and cytokine generation of NK cells in breast cancer as well as thyroid cancer through the EP4 signaling pathway (59, 60). In mouse tumors, NK cell-derived chemokines CCL5 and XCL1 promote the accumulation of dendritic cells (DCs). PGE2-producing tumors inhibit NK cell function, chemokine production, and DCs chemokine receptor expression to promote tumor immune escape (55). Breast tumor cells have been shown to express fewer MHC class I molecules when EP4 signaling is inhibited, which enhances the NK cells’ capacity to fight the tumor (61).

### Dendritic cells

DCs have a unique function in the induction and regulation of innate and acquired immune responses. To direct T cell responses, DCs in the TME collect, prepare, and present tumor-associated antigens on MHC molecules as well as supply co-stimulatory and soluble components (62). In humans, reduced DC numbers and activity affect the prognosis of colorectal cancer patients (63). When patients were stratified based on cDC1-related gene expression, higher cDC1 signaling in tumors was found to be positively correlated with survival (55). However, eight studies have come to the conflicting conclusion that tumor-associated DCs can be a predictor of progressive prognosis in colorectal cancer (64).

PGE2 plays an important role in the control of DCs behaviors include differentiating, producing cytokines, polarizing TH cells, migrating, and maturing (65). In addition, it has been found that inhibiting EP2 and EP4 receptors increases both MHC molecule expression and antigen uptake by lung dendritic cells (11). Tumor-derived prostaglandin analogs inhibited both the aggregation and activation of CD103+ DCs within the tumor, including their capacity to produce IL-12, as well as the expression of type I immune-related markers (10). Secretion of PGE2 may inhibit the capacity of DCs to activate CD8^+^ T lymphocytes, thus promoting T cell exclusion from TME (41). Human DC surface HLA class II antigen expression levels are downregulated by IL-6 through the functions of COX-2, lysosomal protease, and arginase (66). Furthermore, an *in vitro* study demonstrated that NSAIDs prevent DCs from presenting MHC-restricted antigens (67). Taken together, the COX-2-PGE2 signaling pathway promotes tumor immune evasion by inhibiting DC cells.

### CD4^+^ T helper Cells

APCs activate naive CD4^+^ T cells, which then initiate differentiation into various effector T cells during inflammatory and immunological responses to infections and cancer (68). Th1 cells, in general, control more aggressive responses by encouraging cytotoxic immune responses, whereas Th2 cells control less tissue-damaging immune responses (69, 70). As a result of resident tissue cells being stimulated to release chemokines by Th17-associated cytokines, neutrophils and macrophages are drawn to the sites of inflammation. In turn, the recruited cells generate more cytokines and protein hydrolases, exacerbating the immunological reaction (71, 72). TGF-β suppresses TH2-mediated cancer immunity. In a mouse model of breast cancer, induction of TGF-β receptor II gene deletion in CD4^+^ T cells inhibited tumor growth (73). Higher levels of circulating CD4^+^ T lymphocytes were linked to smaller tumor sizes in GC patients (74). Th17 cells were found in greater numbers in the peripheral blood and tumor tissue of patients with oral squamous cell carcinoma (66). In conclusion, the formation and progression of tumors are highly correlated with CD4^+^ T cell activity.

COX-2 deficiency delays mammary carcinogenesis by enhancing type 1 immune responses in breast cancers (75). At high doses, PGE2 induces a shift from a Th1-dominant to a Th2-dominant immune response by downregulating Th1-induced activation of the IL-12 pathway (68). Additionally, PGE2 stimulates IL-23 production and prevents DCs from releasing IL-12 and IL-27, which increases the pathogenic inflammatory Th17 phenotype (76). Although the majority of published studies indicate that PGE2 boosts Th2-type responses, evidence shows that COX-2 has an inhibitory effect on Th2 immune responses. In contrast to Th1-mediated lung inflammation, Jaffar et al. demonstrated that specific inhibition of COX-2 *in vivo* decreases PGE2 production and causes a considerable rise in Th2-mediated lung inflammation (77). Some studies have shown that PGE2 can promote Th1 differentiation through the EP1 receptor (78). Thus, the results are contradictory and more research is required.

### CD8^+^ cytotoxic T cells

CD8^+^ cytotoxic T cells, the primary killer cells of pathogens and tumor cells, are essential for the destruction of intracellular infections and malignant cells, and may provide long-term immune protection (79, 80). Exercise alters the metabolism of CD8^+^ T cells, thereby enhancing their antitumor efficacy (81). In colorectal cancer, low tumor stage, negative nodal stage, longer overall survival, and an inflammatory immune phenotype were all substantially correlated with the density and proportion of proliferating CD8^+^ cytotoxic T cells (82). These results suggest that CD8^+^ T cells are capable of suppressing tumor growth.

In ErbB2 transgenic mice, COX-2 (MEC) KO breast cancer tumors contained more CD8^+^ cytotoxic immune cells (CTL) (7). Some studies found that the amount of CD8^+^ T cells and the percentage of functional CD8^+^ T cells were considerably decreased in PTGS2 overexpression tumors (83). These findings suggest that activation of the COX2 enzymes can reduce tumor-infiltrating CD8^+^ T cells. In addition, effector CD8^+^ T cells in tumors are inhibited from killing tumors by tumor-producing, PGE2-activated immunosuppressive cells in TME *via* EP4 receptors (84). During chronic lymphocytic choroidal meningitis virus infection, EP2 and EP4 are upregulated on virus-specific CTL inhibiting CTL survival and function (85). Increased secretion of PGE2 by breast cancer cells also recruits Treg cells into the primary tumor, thereby increasing apoptosis of CD8^+^ T cells and bone metastasis of cancer cells (86). PGE2 directly inhibits cytotoxic T cell activity and induces regulatory T cell function *in vitro* through upregulation of CD94 and NKG2A complexes (87). However, more evidence is needed to demonstrate whether the COX-2-PGE2 pathway promotes tumor immune evasion by suppressing CD8^+^ T cells.

## Potential strategies for cancer therapy

Immunotherapy has grown in popularity as a cancer treatment option in recent years. Given the importance of the COX-2-PGE2 pathway in carcinogenesis and progression, there is a clear opportunity for therapeutic intervention. NSAIDs are a class of drugs that primarily suppress COX enzyme activity, which also lowers prostaglandins production. The use of NSAIDs to inhibit tumor growth has been considered. Combination therapy with NSAIDs and anti-PD-1 monoclonal antibody induces tumor eradication faster than anti-PD-1 alone (10). In addition, an *in vivo* investigation revealed that NSAIDs improved anti-tumor responses and reversed the imbalance between Th1 and Th2 in the metastatic spread of colorectal cancer (88). Some studies have demonstrated that NSAIDs can reduce cancer-associated mortalities and lower cancer incidence (89). For this reason, the FDA has approved celecoxib, a selective COX-2 inhibitor, for the treatment of population with familial adenomatous polyposis who wish to avoid developing colorectal polyps. In experimental animals, specific COX-2 inhibitors can prevent the development of mammary tumors (90). However, due to the risk of adverse cardiovascular events, celecoxib, along with other NSAIDs (except from aspirin), should not be used for an extended period of time, particularly in patients with a history of atherosclerotic heart disease (91). Excessive bleeding is the most serious side effect of aspirin, and both the risk and mortality rate increase with age (92). One way to prevent these negative consequences is to target only downstream PGE2 signaling. Cell surface inhibitory receptors (such as PD-1) are centrally involved in T cell exhaustion (93). And PGE2 could be one of the inducers of PD-L1 expression (94). We suppose that COX2-PGE2 pathway may result in T cell exhaustion by inducing PD-L1 expression. Emerging data from multiple solid tumor mouse models indicates that EP4 antagonists and PD1/PD-L1 blockade are effective in inhibiting primary tumor growth (49, 95, 96). Therefore, it may be a new potential strategy to combine EP antagonists with PD1/PD-L1 blockade in treating T cell exhaustion. In addition, combination immunotherapy with chimeric antigen receptor T (CAR-T) cells and checkpoint blockade is thought to be the next immunotherapy frontier (97). For instance, combination PD-L1 blockade and CD19 CAR-T cell therapy resulted in better outcomes in patients with heavily B-ALL (98). PGE2 signaling can activate protein kinase A, and then inhibits T-cell receptor activation, which inhibits the antitumor effect of CAR-T cell therapy (99). Thus, we conclude that targeting EP receptors (such as EP2 and EP4) could represent a novel strategy for improving the efficacy of adoptive immunotherapy. Some clinical trials are ongoing to evaluate the efficacy of these inhibitors. A phase I trial showed that EP4 antagonist (E7046) was safe in patients with advanced malignancies (ClinicalTrials.gov, Number NCT02540291). 1 clinical trial is recruiting patients to evaluate whether a dual EP2/EP4 antagonist (TPST-1495) suppresses tumor growth (ClinicalTrials.gov, Number NCT04344795). Although there is abundant EP receptor expression in fibroblasts and inflammatory cells, the role of the COX-2-PGE2 signaling pathway in TME is not fully understood, and more studies are needed to demonstrate this.

## Summary

In this review, we discuss the role of the COX-2-PGE2 pathway in tumor immune evasion regulation. The idea that efficient therapeutic approaches should involve eliminating tumor cells and suppressing tumor immune evasion is being supported by a growing body of evidence, such as the use of checkpoint inhibitors to target immunosuppressive cells and reactivate immunosuppressive effector T cells. The COX-2-PGE2 signaling pathway may assist tumors in evading immune systems by inducing tumor-associated immune cells aggregation, impaired APC activity, a switch from Th1 to Th2 and Th17 immune responses or by suppressing CD8^+^ cytotoxic T cell and NK cell functions to promote tumor immune escape. In conclusion, the COX-2-PGE2 pathway not only is an effective target for tumor eradication, but it also suppresses tumor immune escape. Therefore, the addition of cyclooxygenase-2 inhibitors or EP antagonists to standard treatment may enhance the response of immunotherapy in anti-tumor immune escape.

## Author contributions

KJ and CQ drafted the manuscript and revised the manuscript. JL and BL were in charge of the whole research conduction and paper writing. All authors contributed to the article and approved the submitted version.
